# Genetic kidney diseases: Decoding molecular taxonomies to drive precision nephrology and therapeutic innovation

**DOI:** 10.1016/j.gendis.2026.102226

**Published:** 2026-05-06

**Authors:** Zhi-ying Liu, Xu-jie Zhou

**Affiliations:** a Renal Division, Peking University First Hospital, Beijing 100034, China; b Kidney Genetics Center, Peking University Institute of Nephrology, Beijing 100034, China; c Key Laboratory of Renal Disease, Ministry of Health of China, Beijing 100034, China; d Key Laboratory of Chronic Kidney Disease Prevention and Treatment (Peking University), Ministry of Education, Beijing 100034, China; e State Key Laboratory of Vascular Homeostasis and Remodeling, Peking University, Beijing 100034, China

**Keywords:** Emerging therapies, Genetic kidney disease, Genetic testing, Molecular mechanisms, Precision medicine

## Abstract

Genomic advances have transformed the landscape of genetic kidney diseases, enabling a shift from traditional phenotype-based diagnostics to molecularly precise classification. Large-scale genetic testing has revealed that approximately 10% of chronic kidney diseases are monogenic, greatly improving disease recognition and refining epidemiological prevalence—most notably for autosomal dominant polycystic kidney disease, Alport syndrome, podocytopathies, and autosomal dominant tubulointerstitial kidney disease. Importantly, genetic diagnostics have clarified previously uncertain etiologies in both pediatric and adult populations, enhancing diagnosis, prognosis, and clinical management. Integration of molecular genetics into nephrology has also exposed key limitations of phenotype-driven classification: genotype-phenotype discordance, single-gene variants causing diverse clinical syndromes, and similar phenotypes arising from distinct genetic etiologies. This complexity underscores the risks of relying solely on clinical observation, including inappropriate treatments or missed diagnoses that compromise patient outcomes. For instance, multiple renal cysts can result from various genetic disorders, and misclassification as autosomal dominant polycystic kidney disease may lead to inappropriate tolvaptan use. Molecular diagnostics now enable precision medicine by guiding therapy, optimizing transplantation decisions, and facilitating genetic counseling. Meanwhile, innovative therapies targeting the DNA-RNA-protein-epigenetic axis are advancing toward mechanism-driven treatments that deliver genuine patient benefits. Nonetheless, critical gaps remain in variant interpretation, equitable access to testing, and translation of genetic discoveries to clinical practice. The ongoing evolution of multi-omics integration, robust variant curation, and targeted delivery platforms holds promise for further breakthroughs, transforming outcomes and care standards for patients with genetic kidney diseases and ushering in an era of true precision nephrology.

## Introduction

Genetic kidney diseases represent a diverse and complex spectrum of disorders caused by pathogenic variants that disrupt renal structure and function.^1^^,^^2^ Over the past two decades, this field has been revolutionized by landmark achievements such as the Human Genome Project and the advent of massively parallel sequencing.^2^^,^^3^ These advances in genetic technologies have greatly improved the identification of disease-causing genes, while simultaneously making genetic testing more accessible and affordable in clinical practice. To date, more than 625 genes have been implicated in chronic kidney disease (CKD), underscoring the remarkable genetic heterogeneity of renal pathogenesis and exposing the limitations of traditional phenotype-based classifications.^4^, ^5^, ^6^, ^7^, ^8^, ^9^, ^10^, ^11^, ^12^ Consequently, nephrology has undergone a shift from anatomical or eponymous taxonomies to molecular classifications that more accurately reflect the biological mechanisms of kidney disease.

The incorporation of genetic insights into clinical nephrology has fundamentally transformed patient care. Both pediatric and adult renal disorders are influenced by genetic factors, with varying heritability for traits such as glomerular filtration rate and albuminuria. Monogenic conditions—including autosomal dominant polycystic kidney disease (ADPKD), Alport syndrome, and a range of tubulointerstitial nephropathies—can now be precisely diagnosed using targeted gene panels or exome sequencing, enabling earlier interventions and personalized management strategies.^13^, ^14^, ^15^ In parallel, the discovery of common risk alleles such as apolipoprotein L1 (*APOL1*) has bridged the gap between rare Mendelian diseases and complex polygenic models, shedding light on population-level disparities in CKD prevalence and outcomes.^16^, ^17^, ^18^, ^19^ The implementation of genetic testing in clinical practice has yielded tangible benefits: clarification of uncertain diagnoses, improved prognostic predictions, guidance for treatment selection, informed family counseling, and optimized transplantation planning.^20^ Diagnostic yield is particularly high in pediatric and syndromic cohorts, while ongoing improvements in sequencing technology continue to expand clinical applications.^5^^,^^6^^,^^21^, ^22^, ^23^

The integration of molecular testing has catalyzed a paradigm shift from phenotype-driven diagnosis to molecularly informed taxonomy, leading to four major advances: i) precise diagnostic reclassification, ii) molecular-based prognostic prediction, iii) personalized treatment strategies, and iv) development of gene-targeted therapies. Genetic diagnosis helps clinicians avoid ineffective or potentially harmful treatments, refine decisions related to transplantation, and provide accurate risk assessments to affected families. Moreover, a deeper understanding of molecular mechanisms has supported the development of therapies aimed at key disease pathways, moving beyond symptomatic management toward causative interventions.

Despite these advances, important challenges persist. Variant interpretation, particularly with regard to variants of uncertain significance, remains a critical bottleneck. Disparities in access to testing across geographic and socioeconomic contexts exacerbate health inequities. Furthermore, translating genetic discoveries into approved therapies remains complex and slow. To address these barriers, recent research emphasizes the importance of integrating data from both coding and regulatory regions, as well as mapping genetic perturbations to specific kidney cell types most relevant to disease pathogenesis. These strategies are driving improvements in diagnostic accuracy and accelerating therapeutic innovation, though knowledge gaps remain.^24^, ^25^, ^26^, ^27^, ^28^

This review provides a comprehensive synthesis of the evolution, current landscape, and emerging frontiers in genetic kidney disease research. We highlight how advances in genomics have refined disease classification, improved diagnostic precision, and informed clinical management, while also addressing ongoing challenges and opportunities. Particular attention is given to precision medicine approaches and innovative therapeutic strategies—including RNA-based modulation, gene editing, and kidney organoid models—that target the root causes of genetic kidney disorders and hold promise for transforming future standards of care.

## Historical milestones in human genetics

The modern history of human genetics began with the discovery of the DNA double-helix structure by Watson, Crick, Wilkins, and Franklin in 1953, a seminal event that established the foundation of molecular biology.^29^ The subsequent development of DNA sequencing by Frederick Sanger in 1977 provided the first method for decoding nucleotide sequences, enabling the complete sequencing of small viral genomes and laying the groundwork for the Human Genome Project.^30^ Initiated in 1990 and completed in 2003, the Human Genome Project achieved the first comprehensive map of the human genome, identifying approximately 20,000–25,000 protein-coding genes and over 1.4 million single-nucleotide polymorphisms.^31^ This global endeavor not only provided the genetic blueprint of human biology but also spurred the development of massively parallel sequencing, which reduced the cost and time of genome sequencing by orders of magnitude.^3^^,^^32^ The cost of sequencing an entire human genome has dropped from $2.7 billion in 2003 to less than $1000 today, thereby democratizing access to genomic analysis.^32^

Advances in sequencing technologies, particularly massively parallel sequencing and long-read sequencing, have further expanded the scope of genetic research and clinical applications. Long-read sequencing permits accurate characterization of structural variants and complex genomic regions, while large-scale initiatives such as the UK Biobank, aiming to sequence 500,000 individuals, exemplify the power of population-level genomic studies.^33^ These technological breakthroughs have transformed genetics from a predominantly research-focused discipline into a central pillar of precision medicine, profoundly reshaping the clinical understanding and management of genetic kidney diseases.

## The role of genetics in kidney disease: Enhanced disease recognition and epidemiological insights

Genetic architecture influences kidney disease across a continuum of allele frequencies and effect sizes. To date, more than 625 genes have been associated with CKD, reflecting a striking heterogeneity of renal pathogenesis encompassing monogenic causality, high-impact risk alleles, and polygenic susceptibility.^6^^,^^12^

Monogenic kidney diseases arise from rare, highly penetrant variants in a single gene, sufficient to cause disease on their own. For example, pathogenic variants in collagen IV genes (*COL4A3/4/5*) underlie 30%–78% of familial glomerular diseases such as Alport syndrome and focal segmental glomerulosclerosis (FSGS).^6^^,^^11^^,^^34^ Similarly, ADPKD, caused primarily by variants in polycystin-1/2 (*PKD1* or *PKD2*), represents the most common inherited kidney disease, affecting 1 in 400–1000 individuals worldwide.^35^, ^36^, ^37^

In contrast, polygenic risk arises from the cumulative contribution of hundreds to thousands of common variants. Heritability estimates range between 36% and 75% for glomerular filtration rate (GFR) and 16%–49% for albuminuria.^38^ Polygenic risk scores enable stratification of large populations into tiers of CKD risk, but their predictive precision for individual clinical trajectories remains limited.^39^ For now, polygenic risk scores are more suited to population-level prediction than bedside prognostication.

Bridging these two paradigms are high-effect risk alleles such as *APOL1 G1* and *G2* variants, which are enriched in individuals of recent African ancestry.^40^^,^^41^ These alleles substantially elevate CKD susceptibility and explain a significant portion of population disparities in kidney disease burden, yet disease manifestation often requires additional “second hits” such as infection, environmental exposures, or genetic modifiers. Similarly, uromodulin (*UMOD*) variants illustrate how common polymorphisms in established Mendelian genes can influence tubulointerstitial kidney disease risk at the population level.^42^^,^^43^ Together, these genetic models underscore the need to integrate monogenic, oligogenic, and polygenic frameworks when interpreting kidney disease etiology.

The broad implementation of genetic testing has fundamentally reshaped the epidemiology of CKD. Inherited forms are now estimated to account for at least 10%–15% of early-onset CKD cases, though prevalence in adults has historically been underestimated. Epidemiological studies reveal that ADPKD affects approximately 9.3 per 10,000 individuals and accounts for 5%–10% of end-stage kidney disease (ESKD) in Western populations.^6^^,^^35^^,^^44^^,^^45^ Alport syndrome contributes 1%–2% of ESKD cases, while podocytopathies—including those due to alpha-actinin 4 (*ACTN4*) or nephrotic syndrome type 1 (*NPHS1*) variants—constitute another major category.^46^, ^47^, ^48^, ^49^ Genetic contributions to autosomal dominant tubulointerstitial kidney disease (ADTKD), particularly UMOD-related subtypes, comprise more than 10% of selected CKD cohorts.^50^^,^^51^

Importantly, genetic discoveries have expanded disease recognition beyond classical associations. In ADPKD, novel causal genes such as glucosidase II alpha subunit (*GANAB*) and DnaJ heat shock protein family (Hsp40) member B11 (*DNAJB11*) have been identified, extending the genetic landscape beyond *PKD1* and *PKD2*.^52^^,^^53^ Similarly, continuing efforts have revealed new genes contributing to diverse hereditary nephropathies, illustrating the evolving complexity of inheritance and pathogenic mechanisms.

The diagnostic impact of genetic testing is particularly evident in cases previously deemed idiopathic. Genetic analysis resolves approximately 30%–50% of unexplained pediatric and adult CKD or ESKD cases, providing unequivocal molecular diagnoses unattainable by conventional work-ups.^4^^,^^6^^,^^9^^,^^48^^,^^54^ Clarification of etiology has profound clinical implications: it enables precision in prognostic counseling, informs treatment decisions, including avoidance of ineffective therapies, supports reproductive planning, and facilitates cascade testing of at-risk relatives. In practice, this enhanced disease recognition not only redefines epidemiological prevalence but also directly shapes individualized clinical management and long-term outcome optimization.

## Reclassification of kidney disease

### The genetic contribution to CKD of an unexplained cause

Clarification of terminology: It is essential to distinguish between the general clinical challenge of CKD without a defined cause and the specific endemic entity known as CKDu. In this review, we focus on the former—cases in which standard diagnostic evaluation fails to identify an etiology, now formalized as CKD of unexplained cause (CKDx).^55^ This category applies globally and represents a diagnosis of exclusion. In contrast, CKDu denotes endemic forms of CKD occurring in specific geographic regions (*e.g.*, Central America, Sri Lanka), often linked to environmental or occupational exposures.^56^^,^^57^ Although genetic insights may inform both, the diagnostic framework and likelihood of monogenic etiologies differ. Accordingly, our discussion now emphasizes the genetic underpinnings of the broader, non-endemic CKDx category.

A substantial proportion of patients with CKD lack a clearly defined etiology, making CKDx a persistent clinical and epidemiological challenge. Genetic testing has emerged as a powerful diagnostic tool in this context. Large cohorts demonstrate that monogenic causes account for approximately 6%–30% of adult-onset CKD and up to 30% in pediatric populations. In carefully selected CKDx cohorts, whole-exome or whole-genome sequencing identifies a pathogenic variant in 17%–40% of individuals.^5^^,^^34^^,^^58^

The genetic underpinnings of CKDx are highly heterogeneous, encompassing more than 60 genes implicated in disorders such as Alport syndrome, nephronophthisis, cystic kidney diseases, and inherited tubulointerstitial disorders.^59^^,^^60^ These discoveries frequently lead to diagnostic reclassification. For instance, a patient previously categorized as having idiopathic FSGS may be correctly re-diagnosed with *COL4A*-related nephropathy.^61^ Such reclassification has direct clinical consequences: it avoids ineffective immunosuppressive therapy, enables timely initiation of renin–angiotensin system inhibitors, and provides accurate guidance for genetic counseling.^62^ Thus, molecular testing transforms CKDx from a heterogeneous clinical label into a series of distinct genetic disorders with tailored management strategies.

### Precision reclassification of related disorders

Traditional diagnostic paradigms in nephrology depend heavily on clinical presentation, renal imaging, and histopathology.^63^ However, many kidney diseases share overlapping clinical and morphological features, resulting in diagnostic ambiguity and limited therapeutic precision. Reliance solely on phenotype-based classification often obscures the underlying molecular cause and limits accurate prognostication.

The widespread application of next-generation sequencing has exposed fundamental limitations in these traditional approaches. A striking degree of genotype–phenotype discordance has emerged, characterized by two predominant patterns: i) a single gene giving rise to multiple distinct phenotypes, and ii) identical phenotypes arising from variants in different genes.

One illustrative example is hepatocyte nuclear factor-1 beta (*HNF1B*) (Fig. 1), where pathogenic variants may manifest as renal cysts, early-onset diabetes mellitus, congenital genitourinary tract malformations, or pancreatic anomalies, often leading to initial misclassification as isolated cystic kidney disease.^64^, ^65^, ^66^, ^67^, ^68^ Similarly, *COL4A3/4/5* variants produce a wide clinical spectrum ranging from classic Alport syndrome to FSGS, IgA nephropathy-like phenotypes, or even isolated hematuria, depending on variant type, genetic background, and environmental modifiers.^46^^,^^69^, ^70^, ^71^, ^72^

**Figure 1:**
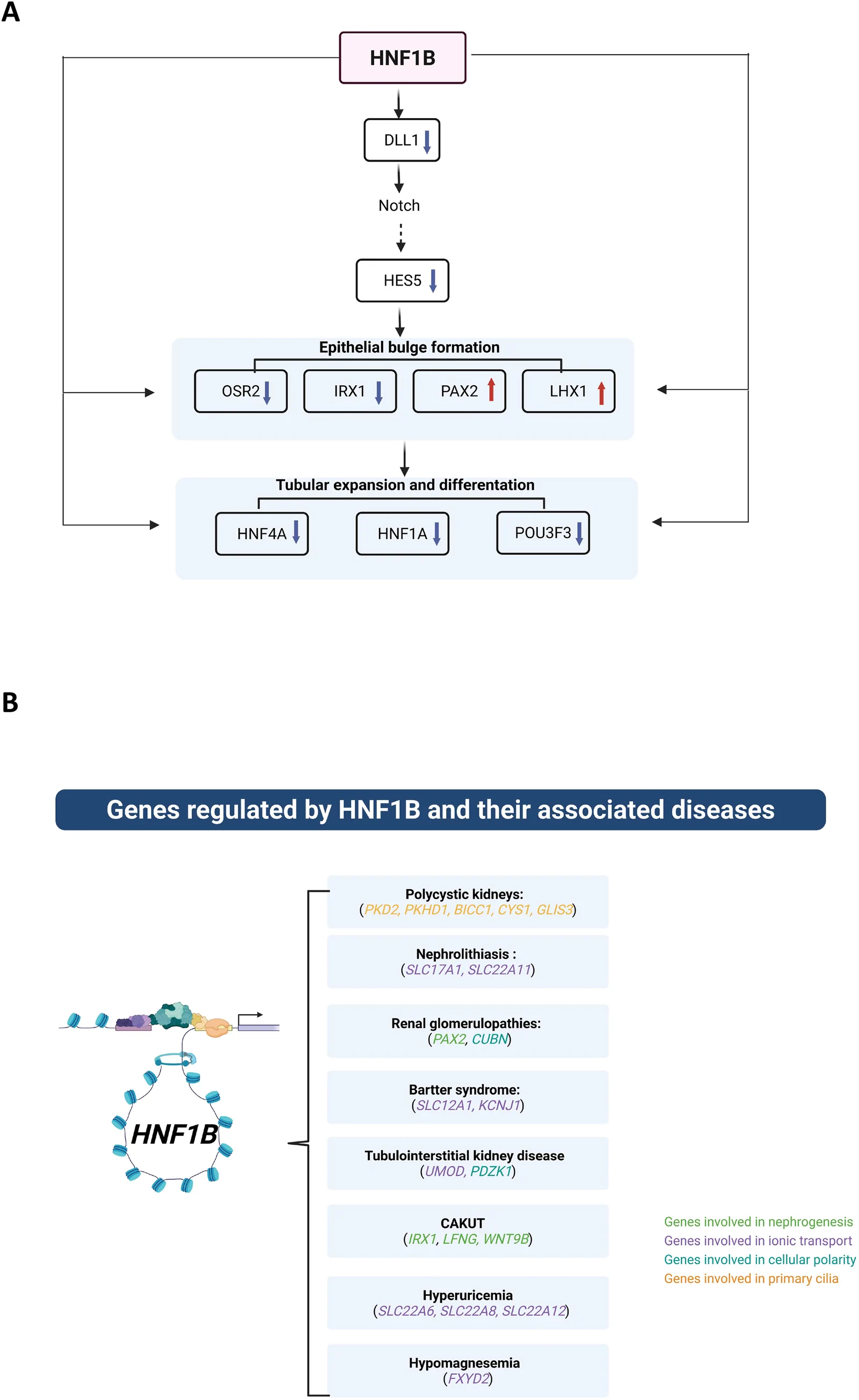
Regulatory network of HNF1B and associated genetic disorders. The schematic focuses on the phenotypic spectrum of HNF1B, demonstrating genotype–phenotype heterogeneity. **(A)** Schematic representation of key genes regulated by HNF1B involved in renal development. The network highlights two major processes: epithelial bulge formation and tubular expansion and differentiation. **(B)** Summary of genes regulated by HNF1B and their association with renal and extrarenal manifestations. Genes involved in nephrogenesis, ion transport, cellular polarity, and primary cilia are shown in green, purple, light blue, and orange, respectively. Renal phenotypes encompass polycystic kidney disease, nephrolithiasis, glomerulopathies, Bartter syndrome, tubulointerstitial kidney disease, congenital anomalies of the kidney and urinary tract (CAKUT), hyperuricemia, and hypomagnesemia. Gene-disease associations are indicated for each category.

Conversely, morphologically similar entities can result from variants in distinct genes. Cystic kidney diseases exemplify this principle: *PKD1/PKD2* variants underlie ADPKD, polycystic kidney and hepatic disease 1 (*PKHD1*) variants cause autosomal recessive polycystic kidney disease (ARPKD), *HNF1B* variants lead to renal cysts and diabetes syndrome, and a wide range of ciliopathy-associated genes contribute additional subtypes.^65^^,^^68^^,^^73^, ^74^, ^75^ Each subtype carries unique prognostic and therapeutic implications, reinforcing the necessity of molecular-based disease classification.

### Redefining FSGS and steroid-resistant nephrotic syndrome

FSGS is among the most common histologic findings in steroid-resistant nephrotic syndrome, representing the third leading cause of ESKD in children and young adults, with a recurrence risk of about 33% following transplantation.^76^, ^77^, ^78^ To date, over 30 monogenic causes of steroid-resistant nephrotic syndrome have been identified, the majority following autosomal recessive inheritance and presenting during childhood, while dominant forms tend to manifest later. Most genes implicated encode proteins crucial for podocyte structure and slit diaphragm function, highlighting podocyte injury as a unifying pathogenic mechanism (Fig. 2).^79^

**Figure 2:**
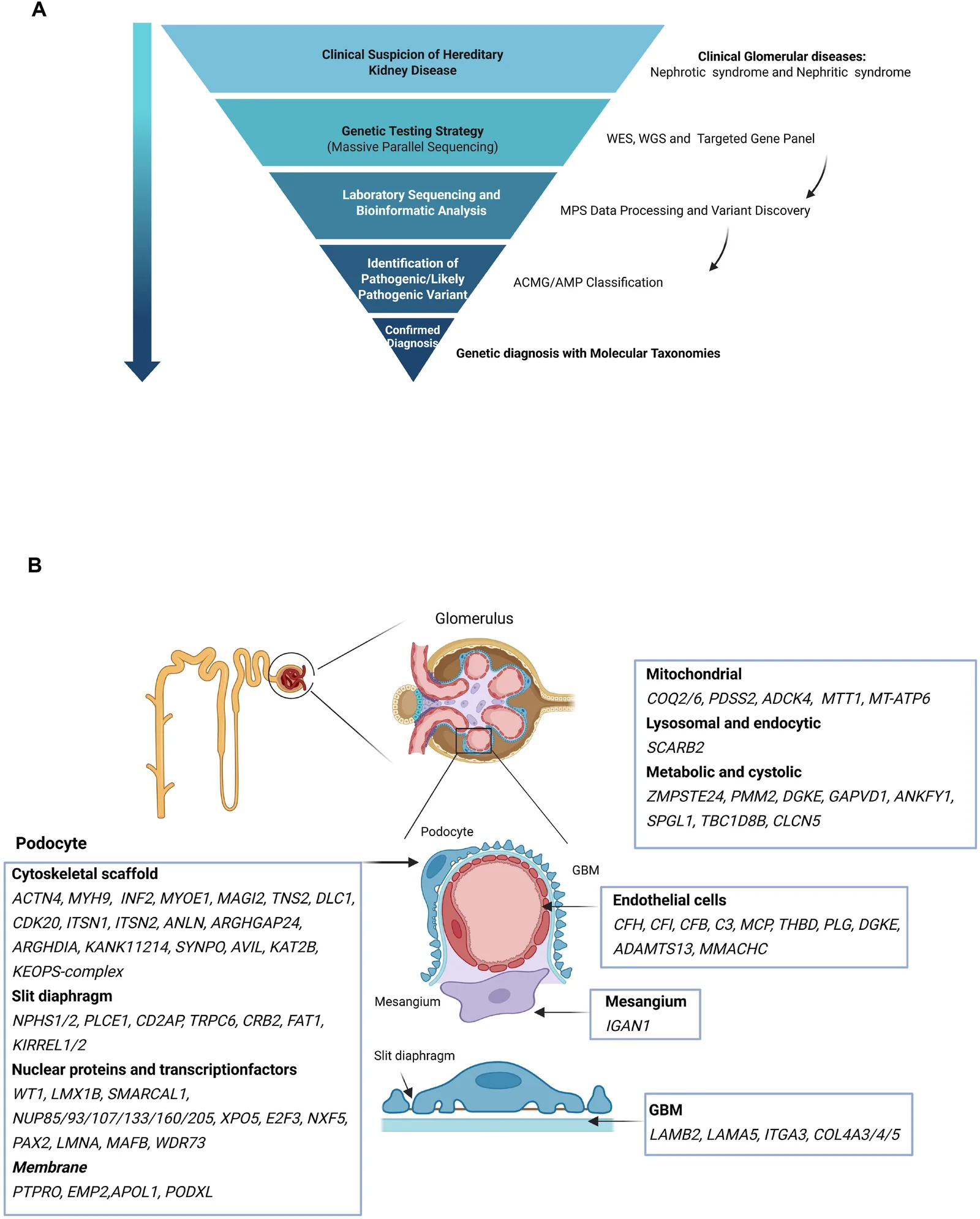
Depiction of genetic testing workflows and genotype–phenotype interpretation challenges, with illustrative examples. **(A)** A clinical diagnostic workflow for suspected hereditary kidney disease. **(B)** Molecular taxonomy of hereditary glomerular diseases. Genes are categorized based on the key glomerular cell types or structures they affect.

For example, *ACTN4* variants cause autosomal dominant FSGS by inducing toxic protein aggregation in podocytes, rendering affected patients entirely resistant to corticosteroids or other immunosuppressants.^80^ Correct genetic diagnosis prevents unnecessary toxicity and guides management toward renin–angiotensin system inhibition and genetic counseling. In contrast, biallelic variants in coenzyme Q10 (CoQ10) biosynthesis genes (*e.g.*, C*OQ2*, *COQ6*, *COQ8B*) produce autosomal recessive FSGS with extrarenal manifestations such as seizures or hearing loss.^81^^,^^82^ Unlike *ACTN4* variants, these subtypes may respond favorably to high-dose CoQ10 (ubiquinone) supplementation, a targeted therapy that can stabilize or even improve renal function.^83^^,^^84^ Additional genetic causes of FSGS include variants in inverted formin 2 (*INF2*) (actin regulation), Wilms tumor 1 (*WT1*) and LIM homeobox transcription factor 1 beta (*LMX1B*) (nuclear transcription factors), and scavenger receptor class B member 2 (*SCARB2*) (lysosomal function), underscoring the molecular diversity underlying this histologic lesion.^85^

### Redefining nephronophthisis as a ciliopathy

Nephronophthisis (NPHP) exemplifies the power of genetic reclassification. Clinically defined by tubulointerstitial nephritis, cyst development, and progression to ESKD, NPHP is now recognized as a prototypical ciliopathy. More than 20 causative genes have been identified, nearly all encoding proteins localized to the primary cilium or its associated structures.^86^^,^^87^ Genetic stratification reveals genotype-specific spectra of extrarenal manifestations. *NPHP1* variants, the most common cause, confer 10%–15% risk of retinitis pigmentosa, warranting routine ophthalmologic screening.^88^ *NPHP3* and transmembrane protein-67 (*TMEM67* or *NPHP11*) variants are strongly associated with hepatic fibrosis and congenital liver disease, necessitating periodic hepatic evaluation.^89^^,^^90^ *NPHP5* (*IQCB1*) and *NPHP6* (*CEP290*) variants cause the Senior–Løken syndrome phenotype, featuring severe early-onset retinal dystrophy.^91^ Variants in *NPHP8* (*RPGRIP1L*), *NPHP9* (*NEK8*), and *TMEM67* cause Joubert syndrome and related disorders, characterized by cerebellar hypoplasia, developmental delay, and unique oculomotor findings.^92^^,^^93^

Disease progression also varies markedly by genotype: for example, *NPHP2 (INVS)* variants cause infantile forms progressing to ESKD within 3–5 years, whereas NPHP1-associated juvenile nephronophthisis typically progresses to ESKD in adolescence.^94^, ^95^, ^96^, ^97^ Determining the precise genetic subtype provides critical prognostic information, guides surveillance for specific extrarenal complications, and enables informed reproductive counseling.

### Molecular taxonomy of genetic kidney diseases

Genetic kidney diseases have traditionally been classified by anatomical involvement (glomerular, tubular, cystic) or by eponymous syndromes, reflecting historical diagnostic limitations (Fig. 3).^98^ However, advances in molecular genetics now enable categorization by genetic mechanisms and protein dysfunction, as exemplified by recent Kidney Disease: Improving Global Outcomes (KDIGO) guidelines for ADPKD and ADTKD.^50^^,^^98^ While these frameworks mark progress, a unified molecular taxonomy remains nascent, necessitating integration of genotypic and phenotypic data to refine diagnostic and therapeutic strategies (Table 1).

**Figure 3:**
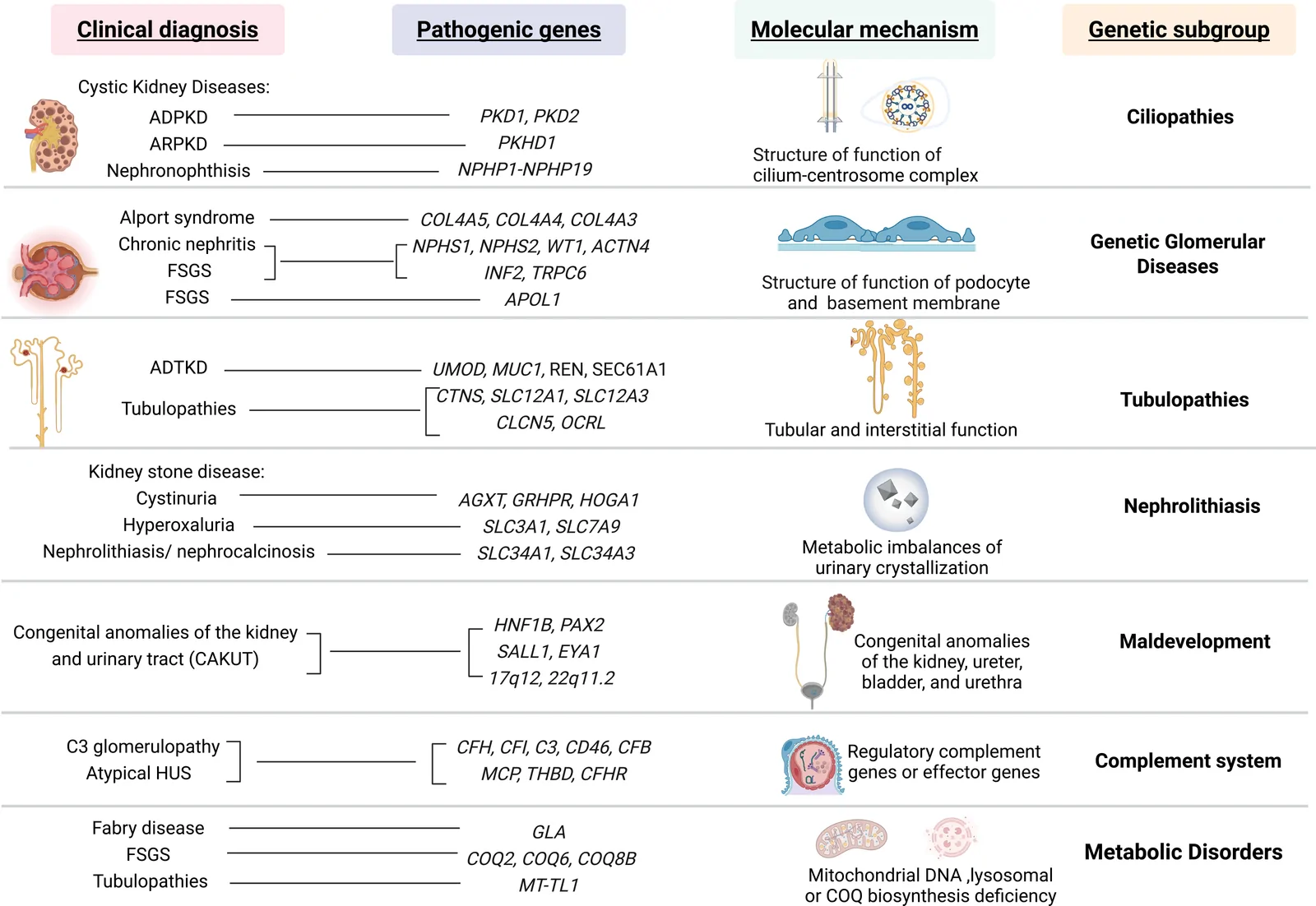
Molecular and cellular classification of hereditary kidney diseases. This gene-based categorization of chronic kidney disease provides critical molecular and mechanistic insights that inform pathophysiology understanding and guide precision treatment approaches. As molecular diagnostics become increasingly integrated into clinical nephrology, this classification system supports the transition toward personalized management strategies tailored to specific genetic etiologies, despite overlapping clinical presentations. ADPKD, autosomal dominant polycystic kidney disease; ARPKD, autosomal recessive polycystic kidney disease; ADTKD, autosomal dominant tubulointerstitial kidney disease; HUS, hemolytic uremic syndrome.

**Table 1:** Representative genotype–phenotype correlations and molecular mechanisms in genetic kidney diseases.

Common clinical features	Genetic kidney disease	Pathogenic genes	Inheritance pattern	Cellular and molecular mechanisms
Cystic kidney diseases	ADPKD	*PKD1*, *PKD2*, *IFT140*, *GANAB*, *NEK8*, *DNAJB11*	AD	Alterations in proteins localized within the cilium–centrosome complex
	ARPKD	*PKHD1*	AR	
	Nephronophthisis	*NPHP1-NPHP19*	AR	
	Bardet–Biedl syndrome	*BBS1–BBS21*	AR	
Genetic glomerular diseases	Steroid-resistant nephrotic syndrome	*ACTN4*, *INF2*, *WT1*, *TRPC6*	AD	Variants in the podocyte-associated genes
		*NPHS1*, *NPHS2*, *PLCE1*, *MYO1E*, *PTPRO*, *GKE*, *ARHGDIA*	AR	
	Fabry disease	*GLA*	XL	Variants lead to α-Gal A deficiency
	Familial amyloidosis	*FGA*, *LYZ*, *APOA1*, *B2M*	AD	Alterations in amyloid proteins
	Nail-Patella syndrome	*LMX1B*	AD	Alterations in type IV collagen of the basement membrane
	Alport syndrome	*COL4A3*, *COL4A4*, *COLA4A5*	AD (AR); AD (AR); XL	
Tubulointerstitial disease	ADTKD	*UMOD*, *MUC1*, *REN*, *SEC61A1*	AD	Variants in ion channels or transporters associated with genes in the tubular
	Renal tubular acidosis	*ATP6V1B1*, *ATP6V0A4*, *SLC4A1*, *SLC4A4*	AR, AR, AD, AR	
	Dent disease	*CLCN5*, *OCRL*	XL	
	Bartter syndrome	*SLC12A1*, *KCNJ1*, *CLCNKB*, *BSND*, *CLCNKA*, *MAGED2*	AR, AR, AR, AR, AR, XL	
	Gitelman syndrome	*SLC12A3*	AR	
	Cystinosis	*CTNS*	AR	
	Liddle's syndrome	*SCNN1G*, *SCNN1B*	AD	
Kidney stone disease	Nephrolithiasis/nephrocalcinosis	*SLC34A1*, *SLC34A3*, *CLDN16*, *CLDN19*, *CASR*, *CYP24A1*	AR	Metabolic imbalances of urinary crystallization inhibitors (*e.g.*, citrate, magnesium) and promoters (*e.g.*, oxalate, calcium, urate, or cystine)
	Cystinuria	*SLC3A1*, *SLC7A9*	AR, AD	
	Primary hyperoxaluria	*AGXT*, *GRHPR*, *HOGA1*	AR	Alterations in genes that are primarily expressed in the liver
Congenital anomalies of the kidney and urinary tract	Congenital anomalies of the kidney and urinary tract	*PAX2*, *HNF1B*, *SALL1*, *EYA1*, *SIX1*, *RET*, *ROMO2*, *UPK3A*, *FGFR2*	AD	Congenital anomalies of the kidney, ureter, bladder, and urethra
Complement-related nephropathies	Atypical hemolytic uremic syndrome	*CFH*, *CFI*, *MCP*, *CFB*, *C3*, *CFHR1*, *CFHR3*, *CFHR4*, *CFHR5*, *THBD*, *DGKE*	AD	Alterations in regulatory complement genes or effector genes
	C3 glomerulopathy			Alterations in complement proteins

Note: This table presents major representative genes associated with hereditary kidney diseases, but it is not exhaustive. Given the rapid discoveries, readers are encouraged to consult up-to-date genetic databases for a complete gene listing. AD, autosomal dominant; AR, autosomal recessive; XL, X-linked; ADPKD, autosomal dominant polycystic kidney disease; ARPKD, autosomal recessive polycystic kidney disease; ADTKD, autosomal dominant tubulointerstitial kidney disease.

The integration of genomics into nephrology has redefined the classification of genetic kidney diseases, shifting from phenotype-based anatomical frameworks to molecularly driven taxonomies.^1^^,^^35^ Over 625 genes are now implicated in CKD, with genetic testing resolving diagnostic ambiguities in 10%–50% of cases and informing personalized management strategies.^1^^,^^8^^,^^11^^,^^23^ Below, we systematically categorize genetic kidney diseases based on genetic mechanisms, clinical manifestations, and recent guideline recommendations.

#### Congenital anomalies of the kidney and urinary tract

Congenital anomalies of the kidney and urinary tract represent the leading cause of pediatric kidney failure, accounting for 40% of childhood ESKD cases.^99^ Genetic drivers include *HNF1B* (5%–15% of cases)^100^ and paired box 2 (*PAX2*) variants, which disrupt renal embryogenesis and often present with renal hypodysplasia, ureteropelvic junction obstruction, or vesicoureteral reflux.^101^^,^^102^ Antenatal ultrasound and postnatal genetic testing are critical for early diagnosis, with multidisciplinary management spanning reconstructive surgery to transplantation.^103^

#### Cystic kidney diseases

ADPKD, caused by *PKD1* (80%) or *PKD2* (15%) variants, remains the most prevalent monogenic nephropathy.^37^^,^^104^ The 2025 KDIGO guideline establishes standardized nomenclature reflecting genetic etiology, recommending formal designations such as *ADPKD-PKD1* (indicating truncating or non-truncating variants) and *ADPKD-PKD2* to tailor clinical management based on genotype. This molecular taxonomy resolves diagnostic ambiguities, as *PKD1*-truncating variants correlate with rapid progression (median ESKD age ∼54 years) compared with *PKD2* (median ESKD age ∼74 years).^98^ Emerging minor genes (*ALG5*, *DNAJB11*, *GANAB*) explain 3%–5% of ADPKD-like cases, often overlapping phenotypically with ADTKD or congenital hepatic fibrosis.^11^^,^^36^^,^^98^^,^^104^ In contrast, ARPKD (*PKHD1*) and syndromic disorders like tuberous sclerosis (*TSC1/TSC2*) highlight the genetic diversity of cystogenesis.^98^^,^^105^^,^^106^

#### Genetic glomerular diseases

Alport syndrome, driven by *COL4A3/4/5* variants, exemplifies molecularly defined glomerulopathies.^107^^,^^108^ X-linked *COL4A5* variants (80% of cases) cause progressive hematuria, proteinuria, and sensorineural hearing loss, while biallelic *COL4A3/COL4A4* variants underlie autosomal recessive forms. Thin basement membrane nephropathy, often from heterozygous *COL4A3/COL4A4* variants, progresses to proteinuria in 20%–30% of patients, blurring distinctions between benign and pathogenic phenotypes.^109^^,^^110^ Genetic testing is now recommended for all unexplained familial hematuria, as 30% of cases initially labeled as idiopathic FSGS or IgA nephropathy harbor collagen IV defects.^111^

#### Tubulointerstitial diseases

ADTKD encompasses *UMOD*, mucin 1 (*MUC1*), *HNF1B*, and renin (*REN*) variants, characterized by progressive fibrosis and bland urinalysis.^50^^,^^112^ ADTKD-*UMOD*, the most common subtype, presents with hyperuricemia and early-onset gout, while ADTKD-*MUC1* requires specialized molecular diagnostics due to cytosine insertion variants.^113^, ^114^, ^115^ While traditionally associated with renal cysts and diabetes syndrome, *HNF1B* variants also cause ADTKD and congenital anomalies of the kidney and urinary tract. This phenotypic variability challenges rigid anatomical categorization, as the same gene can affect glomerular development, tubular function, or systemic metabolism. The 2024 KDIGO CKD guideline now classifies HNF1B-related disease under ADTKD when tubulointerstitial pathology dominates, yet acknowledges its overlap with congenital anomalies of the kidney and urinary tract and cystic disorders.^68^^,^^102^^,^^116^^,^^117^ Kidney biopsy, once central to diagnosis, is increasingly supplanted by genetic testing.

#### Kidney stone disease

Monogenic causes account for 15% of nephrolithiasis cases, with cystinuria (*SLC3A1/SLC7A9*) and primary hyperoxaluria (*AGXT*, *GRHPR*) being prototypic disorders.^118^, ^119^, ^120^ Cystinuria, marked by hexagonal urinary crystals and recurrent staghorn calculi, requires alkalinization and thiol agents to reduce cystine precipitation.^121^ *CLCN5* variants in Dent disease cause proximal tubulopathy with hypercalciuria, while *SLC12A3* defects in Gitelman syndrome lead to hypomagnesemia and hypocalciuria.^122^^,^^123^ Early genetic diagnosis prevents ineffective therapies and guides electrolyte management.

#### Metabolic and lysosomal disorders

Fabry disease, an X-linked lysosomal storage disorder caused by galactosidase alpha (*GLA*) variants, exemplifies systemic metabolic nephropathies.^124^ Deficient α-galactosidase A activity leads to globotriaosylceramide (Gb3) accumulation in podocytes, vascular endothelial cells, and tubular epithelia, manifesting as proteinuria, progressive CKD, and characteristic podocyte vacuolization on biopsy.^125^ Renal involvement often precedes cardiomyopathy or stroke, with males developing ESKD by age 42 on average. Notably, late-onset *GLA* variants (*e.g.*, p.N215S) exhibit milder renal phenotypes but retain cardiovascular risks, necessitating early enzyme replacement therapy or chaperone agents.^126^^,^^127^ CoQ10 deficiencies, such as COQ8B-related nephropathy, disrupt mitochondrial electron transport, presenting with steroid-resistant nephrotic syndrome, FSGS, or tubular dysfunction.^81^^,^^82^

#### Systemic and complement-mediated disorders

Complement dysregulation underlies thrombotic microangiopathies (TMA) and glomerulopathies. Atypical hemolytic uremic syndrome (aHUS), driven by *CFH*, *CFI*, or *CFB* variants, causes endothelial injury via uncontrolled alternative pathway activation, with 50% of patients requiring dialysis.^128^ C3 glomerulopathy (C3G), characterized by dominant glomerular C3 deposition, arises from complement factor H-related 5 (*CFHR5*) variants or anti-C3 convertase autoantibodies, often progressing to ESKD despite immunosuppression.^129^ Genetic testing guides anti-C5 therapies (*e.g.*, eculizumab) in aHUS and identifies monoclonal gammopathy-associated C3G in older adults.^130^^,^^131^

Emerging evidence implicates three-prime repair exonuclease 1 (*TREX1*) variants in renal TMA, expanding the genetic spectrum beyond complement dysregulation. Heterozygous C-terminal frameshift variants (*e.g.*, p.D247fs) in *TREX1*, a DNA exonuclease gene, cause autosomal dominant kidney-predominant TMA without systemic microangiopathic hemolytic anemia.^132^ Unlike complement-mediated aHUS, *TREX1*-related TMA is linked to aberrant type I interferon signaling due to cytosolic DNA accumulation, with renal biopsies showing arteriolar thrombosis and endothelial injury.^133^ Notably, these patients lack response to complement blockade but may benefit from immunomodulatory therapies targeting interferon pathways.

Mitochondrial disorders further blur systemic boundaries, as seen in SRY-box transcription factor 18 (*SOX18*) or tetratricopeptide repeat domain 21B (*TTC21B*) variants causing TMA with neurodevelopmental defects, necessitating genomic retrophenotyping to avoid misdiagnosis.^134^^,^^135^ *TREX1* variants may also manifest as retinal vasculopathy with cerebral leukoencephalopathy, though isolated renal involvement occurs in 15% of cases, emphasizing the need for genetic testing in unexplained TMA.^132^

#### Mitochondrial nephropathies

Mitochondrial nephropathies, caused by mitochondrial DNA or nuclear DNA variants, manifest as tubulopathies, cystic disease, or FSGS. Pathogenic variants in the tRNA Leu (UUR) gene (*MT-TL1*, most commonly the m.3243A>G variant) or COQ biosynthesis genes (*COQ2*, *COQ6*, *COQ8B*) impair oxidative phosphorylation, leading to proximal tubular acidosis, hypomagnesemia, or nephrotic syndrome.^136^, ^137^, ^138^ Heteroplasmy rates in renal cells dictate phenotypic severity, with high mitochondrial DNA variant loads in podocytes correlating with FSGS and tubular dysfunction. Biallelic *COQ8B* variants impair CoQ10 biosynthesis, leading to oxidative stress and podocyte injury, with 50% of untreated pediatric patients progressing to ESKD within 7 months.^83^ Early CoQ10 supplementation can stabilize renal function if initiated pre-ESKD, underscoring the urgency of genetic diagnosis in unexplained proteinuria.^84^ Notably, *COQ8B* variants exemplify the overlap between metabolic and mitochondrial categories, as CoQ10 deficiency disrupts both energy production and redox homeostasis.^139^ Diagnostic challenges include tissue-specific heteroplasmy, necessitating urinary sediment cell analysis when blood testing is inconclusive.

### Significance of genetic research and testing in genetic kidney diseases

Genetic studies and testing have revolutionized the diagnosis, management, and understanding of genetic kidney diseases, offering transformative benefits for patients, families, and clinicians (Fig. 4).

**Figure 4:**
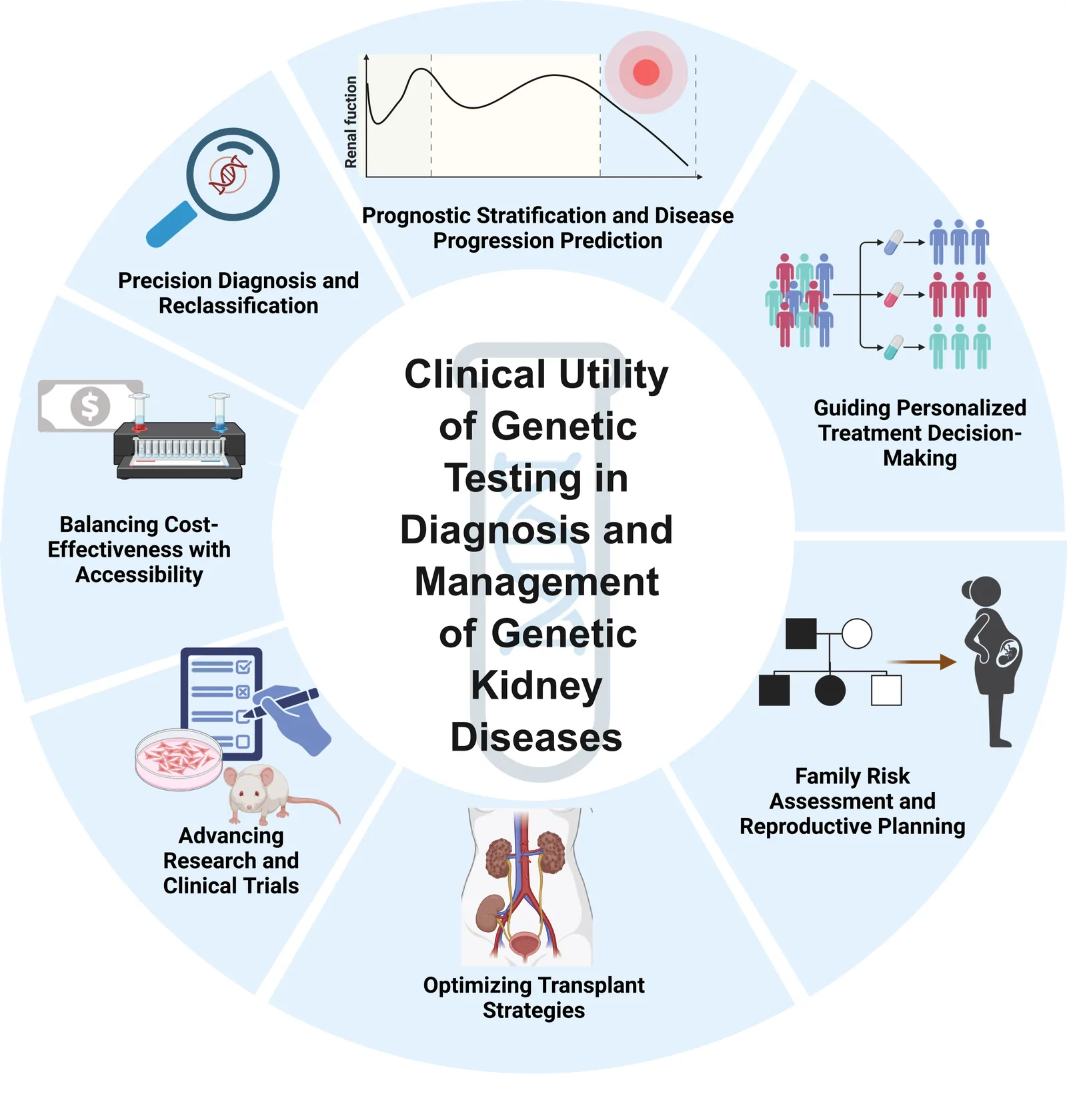
Clinical utility of genetic testing in diagnosis and management of genetic kidney diseases. Illustration of how genetic testing informs diagnosis, prognosis, and individualized patient management.

#### Precision diagnosis and reclassification

Genetic testing resolves diagnostic uncertainty in up to 30% of pediatric and 6%–30% of adult CKD cases.^5^^,^^6^^,^^9^^,^^21^^,^^120^ For example, variants in *COL4A3/4/5* genes can confirm Alport syndrome, distinguishing it from other glomerular diseases, while avoiding invasive biopsies. In cystic kidney diseases, identifying *PKD1/PKD2* variants enables early diagnosis even before radiographic changes. Notably, genetic testing reclassifies initial clinical diagnoses in 20% of cases, refining prognostic accuracy.^6^^,^^11^

#### Prognostic stratification and disease progression prediction

Genetic testing plays a pivotal role in predicting disease progression in genetic kidney diseases, particularly by enabling precise genotype–phenotype correlations that inform clinical management. In ADPKD, as discussed above, patients with *PKD1* variants generally experience a more aggressive disease course and 20-years earlier onset of ESKD compared with those with *PKD2* variants.^36^^,^^140^ Furthermore, the type of variant within *PKD1* significantly affects prognosis: truncating variants (such as nonsense or frameshift variants) are associated with the most rapid decline in kidney function and shortest renal survival, followed by splicing variants, while missense variants tend to confer a milder phenotype and slower progression. These findings are supported by real-world longitudinal studies showing that a *PKD1* truncating genotype, especially when combined with increased kidney size, robustly predicts rapid disease progression and earlier kidney failure.^98^ Similar genotype–prognosis relationships have been observed in other genetic kidney diseases, such as Alport syndrome, where patients with truncating variants in *COL4A3*, *COL4A4*, or *COL4A5* had earlier ESKD than those with missense variants.^108^^,^^141^ Thus, genetic research and testing not only clarify the underlying etiology but also provide actionable prognostic information, enabling risk stratification, individualized monitoring, and timely therapeutic interventions for patients with genetic kidney diseases.

#### Guiding personalized management

Genetic diagnosis directly impacts therapeutic decisions. For instance, detecting *NPHS1/NPHS2* variants in steroid-resistant nephrotic syndrome spares patients from ineffective immunosuppressive therapies.^142^ In aHUS, negative genetic testing for complement pathway variants may justify discontinuing costly anti-C5 therapies.^143^ Additionally, *APOL1* risk allele status informs donor selection in kidney transplantation, balancing familial altruism with long-term donor safety.^144^

#### Family risk assessment and reproductive planning

Identifying pathogenic variants enables cascade screening for at-risk relatives. In ADTKD-*UMOD*, presymptomatic testing allows early intervention in carriers.^145^^,^^146^ Genetic counseling also informs reproductive choices, such as preimplantation genetic testing for families with *COL4A5*-linked Alport syndrome.^108^

#### Transplant optimization

Genetic testing is critical in living-related donor evaluations. Confirming a pathogenic variant in a transplant recipient allows targeted screening of donors to exclude the same variant, particularly in diseases with delayed penetrance like ADTKD-*UMOD*.^147^ For *APOL1*-associated nephropathy, disclosing genotype-dependent ESKD risks (15% lifetime risk) ensures informed donor consent.^148^

#### Advancing research and clinical trials

Genetic data enriches disease-specific registries, uncovering genotype–phenotype correlations and novel therapeutic targets. Patients with monogenic causes of CKD increasingly qualify for gene-targeted trials, such as RNA interference therapies for *APOL1*-mediated disease or small molecules correcting cystic fibrosis transmembrane conductance regulator (*CFTR*) defects.^149^, ^150^, ^151^

#### Cost-effectiveness and accessibility

With out-of-pocket costs below $500 and saliva-based testing options, panels analyzing 400+ kidney-related genes are now widely accessible.^152^ Professional guidelines from the National Kidney Foundation and KDIGO endorse genetic testing as a first-tier tool for undiagnosed CKD, accelerating its integration into routine care.

In China, genetic testing for genetic kidney diseases has become increasingly cost-effective, with prices typically ranging from 2000 to 5000 RMB depending on the scope of analysis. Targeted single-gene tests for specific conditions like Alport syndrome cost approximately 1800 RMB, while comprehensive approaches such as whole-exome sequencing average 4000 RMB or higher. Cost-effectiveness analysis demonstrates that early genetic testing reduces per-patient costs by 20%–41% by avoiding unnecessary biopsies and ineffective therapies. For conditions like familial hypercholesterolemia, lowering genetic testing costs to $38.83 (≈280 RMB) could make population-level screening economically viable.

The clinical value justifies these expenditures: genetic panels analyzing 397 kidney-related genes (*e.g.*, Renasight™) achieve diagnostic rates of 36.9%–67% in pediatric and adult cohorts.^6^^,^^11^^,^^152^ These tests reclassify initial diagnoses in 19%–22% of cases, enabling precision management—for example, avoiding immunosuppressants in *NPHS1/NPHS2*-positive steroid-resistant nephrotic syndrome or guiding *APOL1* risk stratification in transplant decisions. Professional guidelines now endorse genetic testing as first-line investigation for unexplained CKD, accelerating its integration into routine care. With diagnostic yields exceeding 64% in cystic kidney diseases and 70% in adult-onset cases, genetic testing has transitioned from a niche service to an essential nephrology tool.^11^^,^^98^^,^^153^

Accessibility continues to improve through streamlined workflows combining virtual consultations, mobile blood draws, and multidisciplinary interpretation boards. National initiatives and expert consensus further drive adoption, ensuring that this transformative technology reaches both urban centers and rural healthcare networks. Genetic research and testing empower precise diagnostics, tailored therapies, and proactive family management, while driving innovation in nephrology. As testing becomes more affordable and interpretative frameworks mature, its role in reshaping genetic kidney disease care will continue to expand.

### Diagnostic yield of genetic testing in genetic kidney diseases

Genetic testing has emerged as a powerful tool in the diagnosis of genetic kidney diseases, with its efficacy now well established across a range of clinical contexts. In the general population of CKD patients, the overall diagnostic yield of genetic testing varies by age group and clinical presentation. Among unselected adult CKD cohorts, the diagnostic rate for monogenic kidney disease using whole-exome sequencing or targeted gene panels typically ranges from 9% to 25%. For example, a large study of over 3300 CKD or ESKD patients found a genetic diagnosis in 9.3% of cases, with higher rates observed in those with a positive family history or extrarenal manifestations.^6^ In adults with CKD of unknown etiology or suspected hereditary disease, the diagnostic yield increases to approximately 24%–37%, and can be even higher in cases with syndromic features or a strong family history, as demonstrated by studies from both Western and Chinese cohorts.^5^^,^^9^^,^^58^

In contrast, the diagnostic yield in pediatric populations is substantially higher. In children with suspected genetic kidney disease, genetic testing can identify a causative variant in 30%–70% of cases, with the highest rates seen in those with congenital or cystic kidney diseases.^21^ For instance, studies from Chinese multicenter registries report that the diagnostic rate reaches 64% in children with steroid-resistant nephrotic syndrome and up to 80% in those with cystic kidney diseases. The yield is also notable in congenital anomalies of the kidney and urinary tract, with genetic causes identified in approximately 17%–22% of pediatric cases.^116^ These results underscore the greater contribution of genetic factors to early-onset and syndromic kidney diseases compared with adult-onset or sporadic cases.

The clinical context strongly influences the effectiveness of genetic testing. In both adults and children, the presence of a family history of kidney disease, early age of onset, extrarenal features, or rapid disease progression significantly increases the likelihood of identifying a monogenic cause. For example, positive family history increases diagnostic yield to 36%–40% in adults and 46%–58% in children, and cascade testing identifies asymptomatic carriers in 15%–30% of families with *PKD1/PKD2* or *COL4A3/4/5* variants.^11^^,^^98^^,^^153^ In addition, the integration of clinical data—such as imaging findings, renal biopsy results, and detailed phenotyping—can further enhance the interpretation of genetic results and lead to reclassification or clarification of clinical diagnoses. Concurrent hearing loss, ocular abnormalities, or liver cysts boost diagnostic probability for *COL4A5* (Alport syndrome) or *PKHD1* (ARPKD) variants. Neurologic symptoms may indicate *COQ8B*-related CoQ10 deficiency or *MMACHC*-associated cobalamin disorders. For example, genetic testing reclassified or corrected the initial clinical diagnosis in 22% of adult CKD cases, and established a new clinical diagnosis in 38% of cases after whole-exome sequencing analysis.^5^ In addition, genetic testing resolves 38%–48% of cases with unexplained progression, including *APOL1*-associated nephropathy and *INF2*-mediated FSGS.^11^

The choice of testing methodology also impacts diagnostic efficacy. Targeted gene panels tend to outperform whole-exome sequencing in specific clinical scenarios, such as cystic or glomerular diseases, with yields as high as 78% for cystic disease and 62% for glomerular disease.^154^ Reanalysis of negative cases as genetic knowledge expands can further increase diagnostic rates by 10%–15% annually.^152^^,^^155^^,^^156^ Despite these advances, a proportion of genetic kidney diseases remains genetically unsolved, likely due to limitations in current technology, such as the inability to detect non-coding variants, somatic mosaicism, or complex inheritance patterns. Emerging long-read sequencing and functional assays aim to address these gaps.^157^ Standardized variant interpretation frameworks and multidisciplinary boards are essential to mitigate false positives/negatives, particularly in adult populations with age-related confounding factors.^158^

### Clinical and diagnostic evaluation of genetic kidney diseases

The diagnosis and management of genetic kidney diseases require a systematic approach integrating clinical phenotyping, genetic testing, and longitudinal monitoring.

### Comprehensive clinical assessment

The diagnostic process for genetic kidney diseases begins with a thorough clinical evaluation. This includes careful phenotyping, with attention to renal and extrarenal manifestations, age of onset, and disease progression. A detailed family history, ideally spanning three generations, is essential for identifying patterns suggestive of inherited disease. However, clinicians should remain vigilant. While a positive family history strengthens suspicion of genetic etiology, its absence does not exclude hereditary causes, as *de novo* variants or incomplete penetrance occur in 15%–30% of cases.^20^ Clinical assessment is further supported by renal imaging and, where indicated, renal biopsy. Specific findings, such as glomerular basement membrane thinning on electron microscopy, can guide targeted genetic testing for particular conditions like Alport syndrome.

### Indications and strategies for genetic testing

Genetic testing should be considered in patients with early-onset CKD, a positive family history, syndromic features, unexplained CKD with atypical histopathology, or in those being evaluated for transplantation where hereditary disease is suspected.^20^^,^^153^ The diagnostic yield of genetic testing is highest in pediatric populations, where it can reach 30%–70%, and is somewhat lower in adults, typically between 6% and 30%.^4^^,^^6^^,^^21^^,^^58^ In certain clinical scenarios, such as cystic kidney diseases or steroid-resistant nephrotic syndrome, the yield can be even higher. The choice of genetic testing method is influenced by the clinical context; targeted gene panels covering hundreds of kidney-related genes often provide higher diagnostic efficiency for well-defined phenotypes than broader approaches like whole-exome sequencing.^13^^,^^159^

### Genetic counseling and interpretation

Both pre- and post-test genetic counseling are crucial components of the diagnostic pathway. Before testing, patients and families should be informed about the potential outcomes, limitations, and implications of genetic testing, including the possibility of incidental findings. After testing, results must be interpreted in the context of clinical presentation, using internationally recognized guidelines such as those from the American College of Medical Genetics and Genomics (ACMG) and the Association for Molecular Pathology (AMP).^160^ Multidisciplinary teams that integrate clinical, genetic, and laboratory expertise can significantly enhance diagnostic accuracy and inform management decisions. The use of ancestry-matched controls is important for interpretation, as reliance on Eurocentric reference databases may lead to misclassification of variants in underrepresented populations.^161^^,^^162^

### Monitoring and follow-up

A confirmed genetic diagnosis informs a personalized approach to monitoring and follow-up. For example, individuals with truncating *PKD1* variants in ADPKD require earlier and more frequent imaging surveillance than those with *PKD2* variants.^163^ Patients with Alport syndrome benefit from regular audiometric and ophthalmologic evaluations to monitor extrarenal complications. In cases such as *APOL1*-associated nephropathy, genetic findings can influence decisions regarding kidney transplantation, including donor selection. For patients with negative or inconclusive genetic results, periodic reassessment is recommended, as advances in genetic knowledge and testing technologies may eventually yield a diagnosis.^164^

### Treatment of genetic kidney diseases: From conventional management to emerging genetic therapies

The management of genetic kidney diseases has evolved significantly in recent years, integrating traditional CKD therapies with gene-specific interventions and cutting-edge molecular technologies. Elucidation of the molecular basis of monogenic kidney diseases has facilitated the creation of targeted therapies designed to address specific genetic defects and their pathophysiological processes. Thereby, treatment strategies are evolving from symptomatic management to mechanism-based treatment.

### Guiding the use of conventional therapies

Conventional CKD treatment remains foundational and includes renin–angiotensin system blockade with angiotensin-converting enzyme inhibitors or angiotensin II receptor blockers to control proteinuria and slow disease progression; sodium-glucose cotransporter 2 (SGLT2) inhibitors; endothelin receptor antagonists; and mineralocorticoid receptor antagonists, which have demonstrated cardiorenal protective effects across a range of kidney diseases.^165^, ^166^, ^167^, ^168^ Conventional management of CKD has historically focused on slowing disease progression through blood pressure control, proteinuria reduction, and supportive care measures. While these approaches remain valuable for genetic kidney diseases, they fail to address the underlying molecular mechanisms driving disease pathogenesis. More importantly, standard therapeutic protocols may be ineffective or even harmful when applied to specific genetic subtypes without consideration of their distinct pathophysiology.

Genetic testing provides critical guidance for the rational use of conventional therapies, primarily by distinguishing patients who are unlikely to respond to standard interventions from those who may benefit from targeted treatments.^164^ In steroid-resistant nephrotic syndrome, for example, monogenic etiologies are identified in over 30% of pediatric cases.^48^ Patients with pathogenic variants in genes such as *NPHS1/NPHS2* show minimal to no response to immunosuppressive agents, including corticosteroids and calcineurin inhibitors.^169^ Early genetic testing allows clinicians to avoid prolonged, ineffective, and potentially toxic immunosuppressive regimens, shifting management instead to supportive measures such as renin-angiotensin system blockade to reduce proteinuria and slow kidney function decline.^170^ Similarly, in aHUS, the detection of a pathogenic variant in a complement pathway gene (*e.g.*, *CFH*, *CFI*, or *C3*) confirms complement dysregulation as the underlying mechanism and supports the use of terminal complement inhibitors like eculizumab.^171^ Additionally, negative genetic testing for complement pathway variants may justify discontinuing costly anti-C5 therapies. Genetic findings not only help differentiate aHUS from other forms of thrombotic microangiopathy but also inform prognosis and guide decisions regarding treatment duration and discontinuation risk.^130^^,^^172^, ^173^, ^174^

### Optimizing transplantation and family counseling

Genetic testing serves multiple roles in transplantation planning and family management.^15^ In pre-transplant assessment, molecular diagnosis stratifies recurrence risk in the allograft.^77^ Patients with monogenic FSGS, such as those with *NPHS2* variants, have minimal post-transplant recurrence risk.^78^ This contrasts with non-genetic FSGS, where recurrence occurs in 30%–50% of cases due to circulating permeability factors.^78^^,^^175^ In aHUS, genetic findings guide surgical decisions.^176^ Pathogenic variants in liver-expressed complement genes like *CFH* make recurrence nearly inevitable in kidney-only transplants, supporting combined liver–kidney transplantation to address the systemic defect.^177^, ^178^, ^179^

For living-related donor evaluation, genetic testing is essential to mitigate risks of disease transmission and donor morbidity, particularly in autosomal dominant disorders. In ADPKD, targeted testing for *PKD1/PKD2* variants excludes presymptomatic carriers among family members, preventing inadvertent donation from individuals with early cystic changes detectable only via imaging or genetics, thereby safeguarding donor kidney function (with lifetime ESKD risk ∼50% in untreated carriers) and recipient outcomes.^180^^,^^181^ In APOL1-associated nephropathy, prevalent in African ancestry populations, genotyping identifies high-risk genotypes (two risk alleles in 13%–15%), associated with a 15%–20% lifetime ESKD risk and accelerated post-donation estimated GFR decline; this facilitates informed consent, donor exclusion if appropriate, and alternative sourcing, reducing allograft failure rates by 20%–30%.^148^^,^^182^, ^183^, ^184^ For conditions with variable or late-onset penetrance, such as ADTKD due to *UMOD* variants (ADTKD-UMOD), recipient confirmation of pathogenicity enables focused donor testing, avoiding transmission while considering incomplete penetrance (∼70%–90%).^50^^,^^112^ Ethical guidelines emphasize pretest counseling to address potential psychological burdens, such as donor regret or familial discord.^185^^,^^186^

Extending beyond transplantation, genetic testing supports comprehensive family counseling through cascade screening, presymptomatic identification, and reproductive guidance, promoting proactive health management. In families with ADTKD-*UMOD* or mucin-1 kidney disease (ADTKD-*MUC1*), cascade testing detects at-risk relatives with > 90% sensitivity, allowing early monitoring of estimated GFR and interventions like angiotensin-converting enzyme inhibitors to delay progression by 5–10 years.^50^^,^^112^^,^^113^^,^^187^ For X-linked Alport syndrome (*COL4A5* variants), counseling integrates risks of hearing loss and ocular involvement, with presymptomatic testing in females (heterozygous carriers) informing surveillance.^188^^,^^189^ Reproductive options, including preimplantation genetic testing for monogenic disorders like Alport syndrome or ADPKD, enable variant-free embryo selection, reducing inheritance risk to < 1% while respecting autonomy.^159^^,^^190^ Polygenic risk scores, increasingly integrated with monogenic findings, refine counseling by quantifying cumulative CKD susceptibility, though interpretation requires addressing uncertainties in penetrance and gene–environment interactions.^191^^,^^192^ Overall, these applications foster equitable access to care, diminish health disparities, and empower families with evidence-based information, as supported by multidisciplinary guidelines advocating for certified genetic counselors in nephrology clinics.^20^^,^^193^^,^^194^

## Gene- and mechanism-directed therapeutic strategies

### From genetic diagnosis to targeted therapy

The identification of specific pathogenic variants has enabled therapies tailored to molecular lesions, redefining management from symptomatic treatment to genotype-directed intervention.^35^ A compelling example is the distinction between ADPKD and ADTKD, which may both present with cystic phenotypes but require fundamentally different approaches.^112^^,^^195^ Patients with ADPKD caused by *PKD1* or *PKD2* variants benefit from tolvaptan, a vasopressin V2 receptor antagonist shown to slow cyst growth and preserve renal function.^196^^,^^197^ In contrast, patients with ADTKD caused by *UMOD*, *MUC1*, or *REN* variants derive no benefit from tolvaptan; inappropriate use exposes patients to polyuria, nocturia, hepatotoxicity, and healthcare costs without therapeutic gain.

CoQ10 deficiency provides another paradigm of gene-directed therapy. In patients with steroid-resistant nephrotic syndrome due to *COQ2*, *COQ6*, or *COQ8B* variants, early and high-dose CoQ10 supplementation can improve outcomes, reverse nephrotic syndrome, or at least delay progression, by correcting mitochondrial dysfunction and oxidative stress—an approach fundamentally distinct from empirical immunosuppression.^83^^,^^84^ Similarly, for APOL1-associated nephropathy, the small-molecule inaxaplin (VX-147) reduced proteinuria by 47.6% in phase 2a trials by inhibiting the toxic gain-of-function effect of *APOL1* variants, with phase 3 trials underway.^149^ Other precision therapies include transient receptor potential cation channel subfamily C member 6 (TRPC6) inhibitors for TRPC6-mediated FSGS, shown in preclinical studies to reduce podocyte injury and proteinuria.^198^^,^^199^ These approaches exemplify how genetic insights redefine treatment as pathway- or variant-specific.

### RNA-based therapeutics: Modulating gene expression

Beyond DNA-level intervention, RNA-targeted therapies aim to reprogram transcriptional and splicing processes. Antisense oligonucleotides (ASOs) and small interfering RNAs (siRNAs) have demonstrated efficacy in preclinical and clinical models of kidney disease. In Alport syndrome, deep intronic variants in *COL4A5* creating cryptic splice sites were corrected using ASOs, restoring *COL4A5* splicing in patient-derived cells.^200^^,^^201^ Exon-skipping ASOs may convert severe phenotypes into milder forms, while preclinical models showed that ASOs reduced glomerular lesions by 64%.^202^^,^^203^

For siRNA-based therapies, agents such as lumasiran and nedosiran target hepatic enzymes in oxalate synthesis, dramatically lowering urinary and plasma oxalate in primary hyperoxaluria type 1, reducing stone events, and stabilizing renal function.^203^, ^204^, ^205^ These therapies, with proven safety in trials, exemplify how RNA interference can address underlying metabolic defects.

MicroRNA targeting provides another strategy. In ADPKD, overexpression of the miR-17 family aggravates cyst growth; antisense inhibition reduced cyst formation and preserved kidney function in preclinical models.^206^^,^^207^ Together, these RNA-based strategies situate transcriptional and post-transcriptional regulation as a pivotal layer between DNA mutation and protein dysfunction.

### Protein-targeted strategies

At the protein level, therapies aim to compensate for or modulate dysfunctional enzymes and structural proteins. Fabry disease, caused by α-galactosidase A deficiency due to *GLA* variants, illustrates this principle through three complementary approaches: enzyme replacement therapy, pharmacological chaperones, and substrate reduction therapy.^208^, ^209^, ^210^, ^211^, ^212^ Each approach has variant-specific applicability: enzyme replacement therapy provides exogenous enzyme but faces immunogenicity, especially in nonsense variants; migalastat stabilizes misfolded but active enzymes and benefits 35%–50% of patients with amenable variants; lucerastat inhibits upstream substrate synthesis but remains in early testing. Similarly, cystinosis (*CTNS* variants) demonstrates the value of substrate depletion: cysteamine reduces lysosomal cystine accumulation and delays ESKD, although limited by tolerability.^213^^,^^214^

Other protein-directed interventions include stabilizing disrupted collagen IV networks in Alport syndrome and correcting ciliary or cytoskeletal abnormalities in ciliopathies or podocytopathies. These strategies address the protein dysfunction stage of the central dogma, bridging the gap between genetic lesion and cellular phenotype.

### Molecular pathway interventions

Expanding from specific proteins, therapies now target dysregulated molecular pathways. In ADPKD, aberrant cAMP and mTOR signaling drive cystogenesis, supporting targeted use of cAMP modulators, somatostatin analogs, and mTOR inhibitors.^215^, ^216^, ^217^ In ciliopathies like nephronophthisis, recognition of defective ciliary signaling has guided the search for cilia-modulating compounds. Similarly, in Alport syndrome, stabilization of collagen IV scaffolds represents a strategy to reinforce extracellular matrix networks.^217^ Pathway-directed interventions thus harness systems-level insights to counteract the downstream consequences of genetic lesions.

### Direct genetic correction: Gene therapy and genome editing

Perhaps the most transformative line of innovation involves correcting defects at the DNA level. Viral-mediated gene therapy using adeno-associated virus (AAV) vectors and minigene constructs has restored PKD1 expression in PKD models, delaying cystogenesis and renal decline.^218^^,^^219^ Clustered Regularly Interspaced Short Palindromic Repeats (CRISPR)/CRISPR-associated protein 9 (CRISPR/Cas9)-mediated editing has corrected *PKHD1* variants in ARPKD organoids, reducing biliary malformations by 91%, as well as *COL4A5* variants in Alport organoids, normalizing basement membrane expression.^220^, ^221^, ^222^ Further refinement with base editors and prime editing offers greater precision and reduced off-target effects, particularly suited for monogenic kidney disorders driven by point variants.^223^, ^224^, ^225^

Challenges remain substantial: kidney-specific delivery is inefficient, long-term safety requires validation, and ethical boundaries around germline versus somatic editing demand regulatory clarity. Nonetheless, genetic correction illustrates the potential to move from disease modification to true molecular cure.

### Beyond the central dogma: Epigenetic and regenerative strategies

Emerging therapies extend beyond DNA–RNA–protein interventions to encompass epigenetic and cellular layers. PROTACs enable the selective degradation of pathogenic proteins, while mRNA therapeutics aim to transiently restore protein expression without permanent genome editing.^226^^,^^227^ Epigenetic modulation, including targeting of chromatin regulators and DNA methylation, offers novel strategies to reverse maladaptive gene regulation in CKD pathways independent of DNA variation.

Meanwhile, cell-based therapies and kidney organoids represent a frontier of regenerative nephrology. Patient-derived induced pluripotent stem cells allow disease modeling and drug screening, while engineered organoids provide experimental platforms for gene correction and potentially functional replacement.^228^ Although translation faces obstacles of delivery, scalability, and safety, these approaches embody the integration of genetics, epigenetics, and regenerative biology in future kidney care.

### Toward precision nephrology

Together, these strategies illustrate how the therapeutic landscape of genetic kidney diseases is being reconstructed along the continuum of the central dogma—DNA correction, RNA modulation, protein targeting—and extended beyond it through epigenetic and regenerative approaches. Precise molecular diagnosis not only informs eligibility for these therapies but also prevents ineffective or harmful interventions. While challenges remain in delivery efficiency, long-term safety, equitable access, and regulatory implementation, the trajectory of innovation signals a fundamental paradigm shift: from symptom management to molecular cure, ushering in an era of true precision nephrology (Fig. 5).

**Figure 5:**
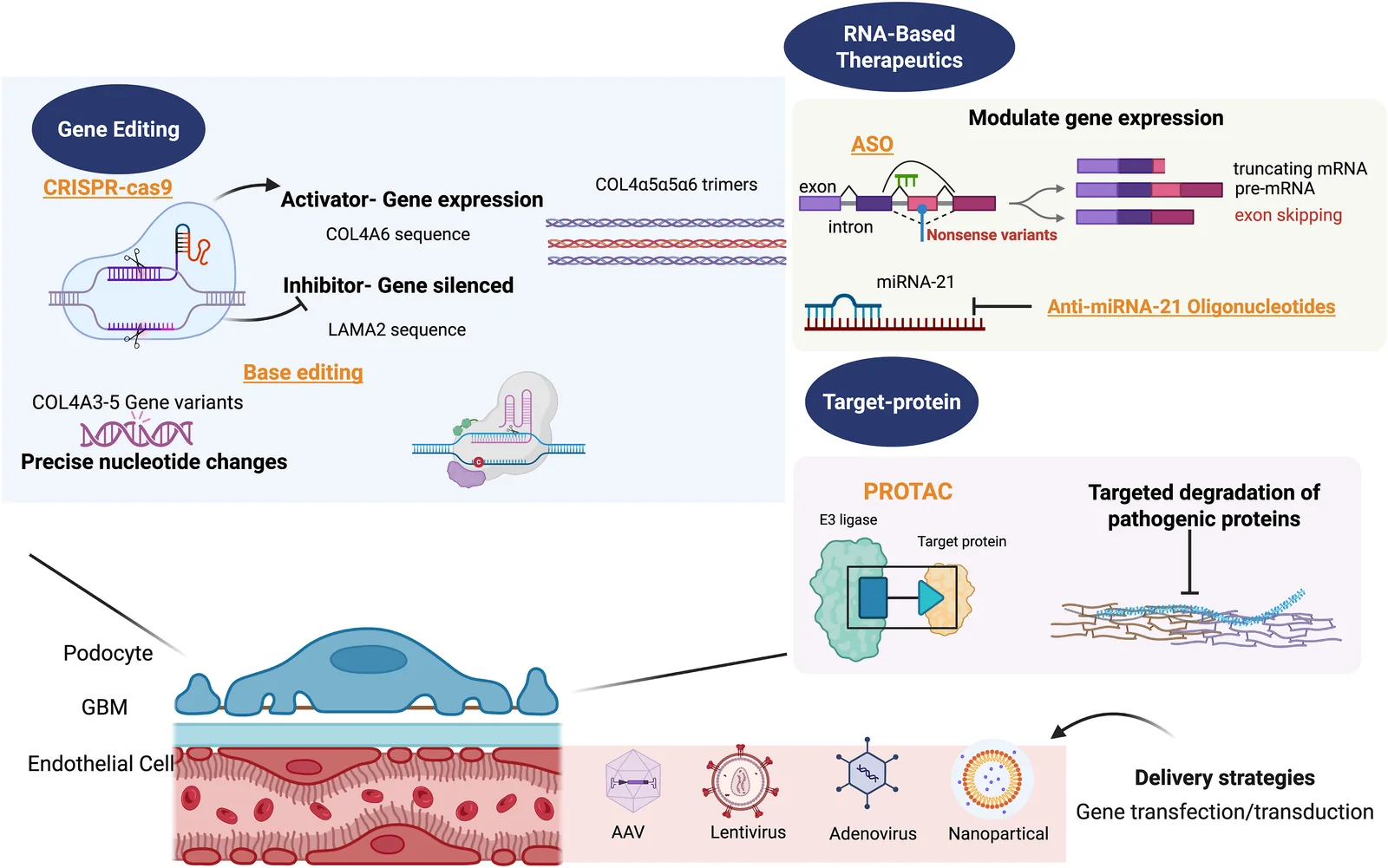
Advanced therapeutic approaches for hereditary kidney diseases. The schematic illustrates three major therapeutic approaches: Gene editing (left panel), RNA-based therapeutics (upper right panel), and target-protein modulation (lower right panel). The lower portion depicts various delivery strategies for these therapeutic agents, including viral vectors (AAV, lentivirus, adenovirus) and non-viral approaches (nanoparticles) that facilitate gene transfection/transduction to specific renal cell types.

## Challenges, opportunities, and future directions in genetic kidney diseases

### Diagnostic and therapeutic challenges

Despite remarkable advances, major obstacles remain in the diagnosis and treatment of genetic kidney diseases. Approximately 15%–30% of cases remain unsolved, often due to pathogenic variants in non-coding regions, somatic mosaicism, or oligogenic inheritance that elude current sequencing approaches.^229^^,^^230^ Phenotypic overlap across diseases further complicates recognition; for instance, COL4A3/4/5-related nephropathy is frequently misclassified as hypertensive nephrosclerosis or IgA nephropathy unless genetic testing is performed.^72^^,^^231^ Even when causal variants are identified, 30%–40% fall into the category of variants of uncertain significance, a problem exacerbated in underrepresented populations lacking ancestry-matched reference databases. Variant interpretation thus remains one of the most significant bottlenecks. On the therapeutic side, animal models often fail to replicate human disease accurately—murine models of ADPKD, for example, do not display the progressive cyst expansion observed clinically, limiting translational relevance.^232^^,^^233^

Economic and infrastructural limitations also restrict the reach of genetic medicine. While sequencing costs in China have decreased to 2000–5000 RMB, rural and under-resourced regions lack facilities for testing, counseling, or multidisciplinary genomic interpretation boards. For ultra-rare diseases such as COQ8B-related nephropathy, delayed diagnosis can result in irreversible renal damage, illustrating the urgent need for more affordable diagnostics and potential newborn screening programs to facilitate timely intervention.

### Gene therapy: Delivery barriers and emerging solutions

Gene-directed therapy remains a promising but technically difficult frontier, with kidney-targeted delivery representing a fundamental challenge. AAV vectors exhibit safety and stable expression but limited renal tropism with systemic administration.^234^^,^^235^ Novel approaches are being developed to improve delivery specificity. Capsid modification, as in AAV-PHP.eB and directed-evolution platforms, has improved renal tubular transduction in experimental models.^235^^,^^236^ Ligand conjugation strategies, such as attaching vectors to folate for proximal tubules or antibodies targeting podocytes, have enhanced tissue specificity.^237^ Direct intrarenal delivery, while bypassing systemic barriers, raises feasibility and safety challenges for routine clinical translation.^238^ Overcoming these delivery barriers will be critical for transitioning gene therapy from experimental use into clinical nephrology.

### Functional genomics and variant interpretation

High-throughput genomics tools are providing unprecedented opportunities to clarify variant function and accelerate therapeutic discovery. CRISPR-based screening platforms allow systematic evaluation of variant pathogenicity in disease-relevant cells, while kidney organoids derived from patient induced pluripotent stem cells provide physiologically relevant systems for variant validation and drug testing.^239^^,^^240^ Multiplexed assays of variant effect (MAVEs) can test thousands of variants within the same gene simultaneously, generating deep functional maps of variant consequences—technologies that have transformed clinical interpretation in *BRCA1* and *TP53* and could do the same for kidney disease genes.^241^^,^^242^

New genome-editing platforms such as base editors and prime editors further enrich this toolkit. Unlike CRISPR nucleases that induce double-strand breaks, base and prime editing permit precise replacement of nucleotides, enabling researchers to model patient-specific variants or test therapeutic edits in cellular models with higher fidelity and lower mutagenic risk.^224^ By combining these technologies, the nephrology field is approaching a future where functional validation and therapeutic evaluation of variants can occur in parallel, shortening the translational gap.

### Systems medicine and multi-omics integration

The future direction of genetic kidney disease research lies in multi-layered integration of genomics, transcriptomics, proteomics, metabolomics, and functional models. Long-read sequencing now detects structural variants in *PKD1/PKD2* that were missed by short-read techniques, increasing diagnostic yields in cystic kidney disease from 64% to 78%.^243^ Patient-derived organoids have highlighted specific mechanisms—for example, HNF1B-deficient organoids demonstrate tubulogenesis defects underlying congenital anomalies.^244^^,^^245^ Coupled with high-throughput drug screening, these models have identified candidate treatments, such as mTOR inhibitors for ADPKD and CFTR modulators for cystic fibrosis-associated kidney disease.^246^^,^^247^

Multi-omics strategies provide broader resolution of disease biology: proteomics can reveal altered signaling cascades invisible at the DNA level, while metabolomics detects biomarkers that track progression or therapeutic response.^248^^,^^249^ However, integrating these heterogeneous datasets remains technically complex, hampered by the lack of standardized pipelines across institutions.^250^ Progress will require clinically deployable bioinformatics tools accessible to non-specialist physicians.^251^

Artificial intelligence offers new solutions by integrating disparate data layers. Machine learning algorithms, including graph neural networks, can model dynamic interactions between genes, proteins, and metabolites to predict disease trajectories or drug responsiveness.^252^^,^^253^ The concept of “digital twins”—computational avatars of individual patients that incorporate genomics, imaging, physiology, and biomarkers—is emerging as a transformative tool for personalized prediction and therapy selection.^254^^,^^255^ Although still experimental, early feasibility data highlight the potential for digital twins to revolutionize management of monogenic kidney diseases.

### Translational and ethical considerations

The translation of these advances faces biological, systemic, and ethical barriers. Drug delivery to kidney tissue remains inefficient: current intravenous siRNA therapies result in only 5%–10% accumulation in renal cells, requiring complex solutions such as nanoparticle formulation or regional administration via retrograde ureteral infusion.^256^^,^^257^ Prenatal and germline gene-editing raise particularly sensitive ethical dilemmas, especially for late-onset conditions such as ADPKD, where risk-benefit assessments remain unresolved.

Global collaboration is hindered by fragmented data infrastructures and variable clinical phenotyping. Pediatric registries like the China Children Genetic Kidney Disease Database (CCGKDD) represent important progress, yet adult cohorts remain inconsistent, limiting large-scale hypothesis testing. Intellectual property disputes surrounding CRISPR technologies and the high cost of novel therapies, exemplified by Zolgensma® at $2.1 million per treatment, raise critical equity concerns for resource-limited settings.^258^ Ethical frameworks, standardized registries, and international collaborations will therefore be essential to ensure that the benefits of genetic nephrology are accessible worldwide.

## Future perspectives

The coming decade is expected to bring transformative advances in the management of genetic kidney diseases, driven by converging innovations in population screening, curative gene therapies, and artificial intelligence. Population-scale genetic programs may enable early identification of high-risk genotypes—for example, *APOL1* risk alleles in individuals of African ancestry—and facilitate the deployment of preemptive interventions such as angiotensin-converting enzyme inhibitors, which have been projected to reduce the incidence of ESKD by up to 30%.^259^^,^^260^ In parallel, breakthroughs in *in vivo* gene-editing technologies are moving closer to clinical translation. AAV-KP1 vectors have achieved nearly 90% podocyte transduction in primates, laying the scientific foundation for *PKD1* minigene therapies aimed at halting cyst growth and preserving renal function. Alongside these therapeutic strategies, artificial intelligence promises to reshape variant interpretation. Deep learning approaches that analyze three-dimensional protein structures and splicing networks are predicted to resolve as many as half of the current variants of uncertain significance by 2030, a development that will significantly strengthen diagnostic precision.

Realizing this potential, however, requires coordinated international efforts to address persistent challenges. Standardized variant curation frameworks must incorporate diverse ancestral backgrounds to mitigate interpretation bias and deliver equitable clinical value across populations. The expansion of human-induced pluripotent stem cell-derived biobanks that represent global genetic diversity will accelerate disease modeling, functional validation, and therapeutic discovery. Ensuring equitable access to emerging molecular therapies remains an urgent priority, necessitating innovative funding and distribution models for orphan drugs and the optimization of kidney-targeted delivery systems. As genetic testing becomes increasingly integrated into routine nephrology practice, bridging the gap between diagnostic capacity and therapeutic innovation will be essential to reducing the global burden of genetic kidney diseases and to establishing a framework of personalized, causality-driven care.

## Conclusion

In summary, the integration of genetic research into nephrology has redefined our understanding and management of genetic kidney diseases across four pivotal domains: precise molecular diagnosis, refined risk stratification, individualized therapeutic decision-making, and the development of gene- and mechanism-targeted treatments. Advances in sequencing technologies have revealed the genetic basis of a significant proportion of early-onset and familial CKD, establishing molecular testing as central to modern nephrology practice. The advent of massively parallel sequencing now enables comprehensive detection of pathogenic variants across a wide spectrum of renal disorders, transforming previously idiopathic cases into clinically actionable diagnoses.

These achievements, however, are counterbalanced by ongoing challenges. Variant interpretation remains a major limitation, particularly in underrepresented populations; equitable access to diagnostic and therapeutic innovations is far from universal; and the clinical translation of emerging genetic and molecular therapies continues to face biological, logistical, and ethical barriers. Addressing these challenges requires standardized genetic evaluation, expanded multi-ethnic resources, functional genomics platforms, and the development of kidney-specific delivery technologies.

The future of nephrology lies in the seamless integration of multi-omics, computational intelligence, and molecular therapeutics into clinical care. As these advances converge, they promise not only to improve outcomes but also to transform genetic kidney diseases from irreversible conditions into disorders amenable to mechanism-based, durable interventions. The field is thus entering an era of true precision nephrology—an era defined by the capacity to intervene at the level of genetic causality, to anticipate disease trajectories, and ultimately to improve the lives of patients and families worldwide.

## CRediT authorship contribution statement

**Zhi-ying Liu:** Writing – review & editing, Writing – original draft, Visualization. **Xu-jie Zhou:** Writing – review & editing, Writing – original draft, Resources, Project administration, Funding acquisition, Conceptualization.

## Funding

The work on this article was supported by the 10.13039/501100012166National Key Research and Development Program of China (No. 2024YFC2511000), the 10.13039/501100001809National Science Foundation of China (No. 82570833, 82370709), the National High Level Hospital Clinical Research Funding (Interdisciplinary Clinical Research Project of Peking University First Hospital; No. 2024IR35, 2022CR41), the 10.13039/501100014764China International Medical Foundation (No. Z-2017-26-2202-2), and the Beijing Bethune Charitable Foundation (China) (No. YPZ-41). The funders had no role in study design, data collection and analysis, decision to publish, or preparation of the manuscript.

## Conflict of interests

The authors declared no potential conflict of interests.
